# The delta neutrophil index (DNI) as a prognostic marker for mortality in adults with sepsis: a systematic review and meta-analysis

**DOI:** 10.1038/s41598-018-24211-7

**Published:** 2018-04-26

**Authors:** Chiwon Ahn, Wonhee Kim, Tae Ho Lim, Youngsuk Cho, Kyu-Sun Choi, Bo-Hyoung Jang

**Affiliations:** 1 Department of Emergency Medicine, Armed Forces Yangju Hospital, Yangju, Korea; 2 Department of Biomedical Engineering, Graduate School of Medicine, Hanyang University, Seoul, Korea; 3 Department of Emergency Medicine, College of Medicine, Hallym University, Chuncheon, Korea; 4 Department of Emergency Medicine, College of Medicine, Hanyang University, Seoul, Korea; 5 Department of Neurosurgery, College of Medicine, Hanyang University, Seoul, Korea; 6 Department of Preventive Medicine, College of Korean Medicine, Kyung Hee University, Seoul, Korea

## Abstract

We performed a meta-analysis to seek evidence for the usefulness of the delta neutrophil index (DNI) as a prognostic blood biomarker for mortality in the early stage of sepsis in adults. A literature search was performed using criteria set forth in a predefined protocol. Studies of adults with sepsis that provided a DNI measurement and that had mortality as the outcome, were included. Review articles, editorials, and non-human studies were excluded. The methodological quality of identified studies was assessed independently by two authors using the Quality in Prognosis Studies (QUIPS) tool. A total of 1,822 patients from eleven studies were ultimately included. Standardized mean differences between non-survivors and survivors were compared. An elevated DNI was associated with mortality in patients with sepsis (standardized mean difference [SMD] 1.22; 95% confidence interval 0.73–1.71; I^2^ = 91%). After excluding two studies—one that included paediatric patients and one with a disproportionately low mortality rate—heterogeneity was minimized (SMD 0.74, 95% confidence interval 0.53–0.94; I^2^ = 43%). Overall, the findings suggest that high DNI values are associated with mortality in septic patients.

## Introduction

Sepsis is a rapidly progressive, life-threatening disease. Accurate and expeditious assessment of sepsis is important for early administration of antibiotics and removal of the source of infection^1,2^. In the 2016 version of the sepsis guidelines (Sepsis-3)^3^, the concept of the systemic inflammatory response syndrome has been deleted. However, it is important for clinicians to distinguish sepsis from a non-infectious inflammatory response in order to institute appropriate treatment; this requires reliable diagnostic tools that reflect early changes^4,5^. Hence, many clinicians have studied the usefulness of blood biomarkers such as C-reactive protein, procalcitonin, and lactate for early assessment of sepsis and for prognostication, in order to initiate timeous treatment and to prevent rapid progression to multi-organ failure^6–10^.

In infectious conditions, mature segmented neutrophils normally proliferate to kill bacteria in the host. Simultaneously, the number of circulating immature neutrophils increases; however, these can cause organ failure in the host^11^. The increase in the number of circulating immature granulocyte is referred to as ‘a left shift’, defined as an elevated immature/total granulocyte ratio or an elevated neutrophil band count^12,13^. In sepsis, this reflects severity and aggravation of the disease course^14,15^. Although accurate measurement of immature neutrophil numbers is needed as a blood biomarker, practically, such quantification is not readily accessible^16,17^. The delta neutrophil index (DNI) is the immature granulocyte fraction provided by a blood cell analyser; it is determined by subtracting the fraction of mature polymorphonuclear leukocytes from the sum of myeloperoxidase-reactive cells and reflects the number of immature neutrophils as a blood biomarker. This index is calculated by differentiating two granulocyte measurements; one measured using the cytochemical myeloperoxidase reaction and the other by the nuclear lobularity channel^18^. Since measurement of the DNI is reproducible, rapid, and accurate, the DNI has been used as a blood biomarker in patients with sepsis. This systematic review aimed to seek evidence for the usefulness of the DNI as a prognostic blood biomarker of mortality in patients in the early stage of sepsis.

## Results

### Study selection and characteristics

**Figure 1:**
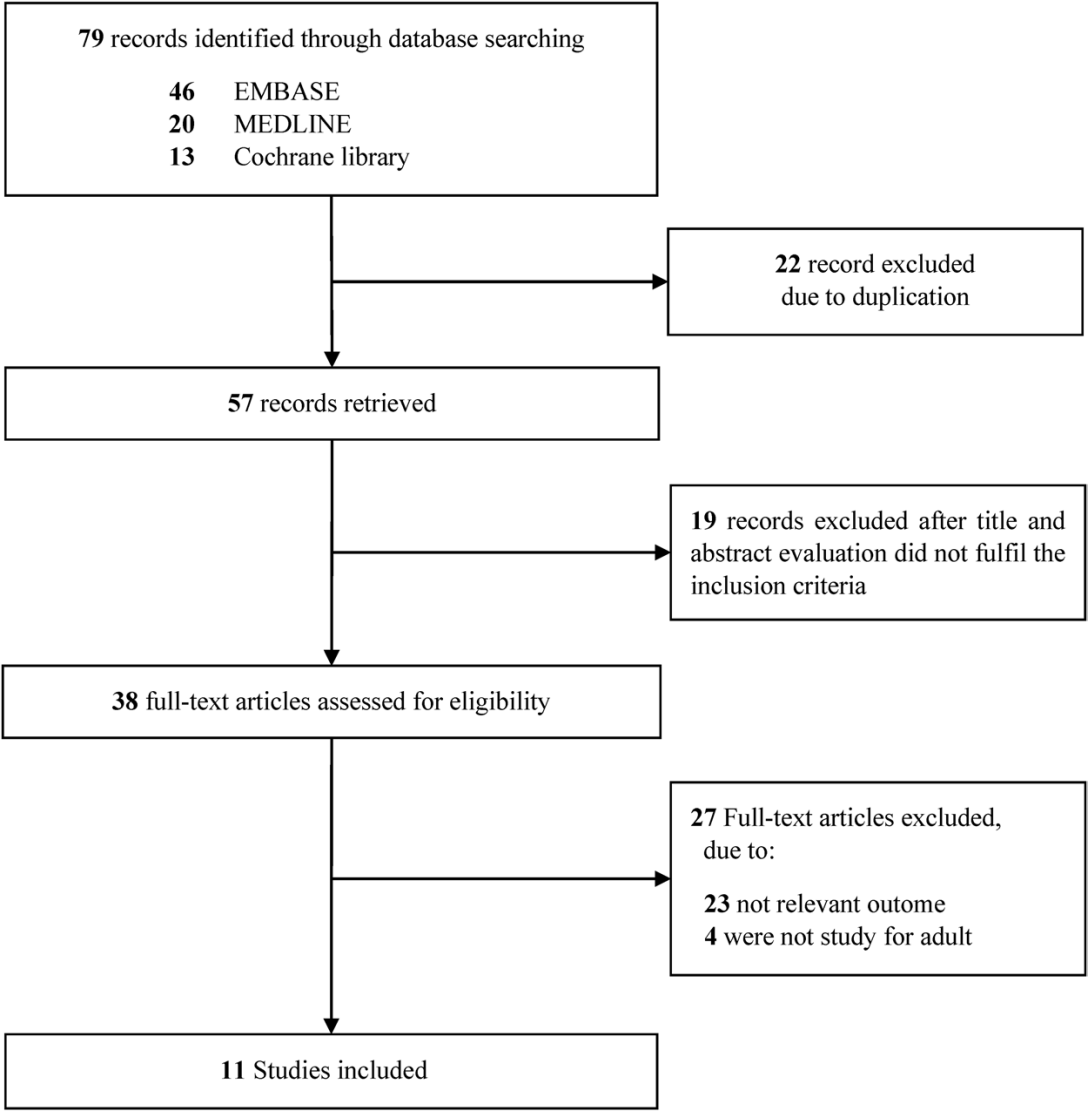
Flow chart of the study selection process.

The process for identifying eligible studies is shown in Fig. 1. Searches of the databases identified 79 articles. A total of 57 studies remained after excluding duplicate articles. Of these, 19 articles were excluded because their titles and abstracts did not fulfil the inclusion criteria at initial screening. The full texts of 38 potentially relevant studies were comprehensively reviewed. Of these, 29 articles were excluded for the following reasons: non-relevant outcome or non-adult study population. Finally, eleven studies met the criteria and were included in the review; nine were full publications^17–25^ and two were abstract-only publications^26,27^.

The main characteristics of the eleven eligible publications are shown in Table 1. In addition, details of the population included in each study are provided in Supplementary Table S1. All included studies were observational, were about sepsis, and had mortality as the main outcome. Ten studies were conducted in Korea, one was conducted in Egypt. Three studies had as their inclusion criteria, cases of bacteraemia. Eight studies included patients with sepsis or septic shock while the three remaining studies had subjects with acute kidney injury, acute cholecystitis, and spontaneous bacterial peritonitis, respectively. In seven studies, the time that blood was sampled to measure the DNI was exactly stated; this detail was unclear or not reported in the other studies. In seven studies, 28-day or 30-day mortality was assessed, whereas two studies assessed 10-day or in-hospital mortality. In these studies, the mean ± standard deviation (SD) of the DNI value (%) was measured by univariate analysis comparing survivors and non-survivors. The standardized mean difference (SMD) of the DNI values was calculated, with the 95% confidence interval (CIs).

**Table 1:** Details of identified studies.

Study identification	Location	Inclusion period	DNI measurement devices	Number of subjects	Inclusion criteria	Age (year)^a^		Male, %		Time of DNI measurement	Mortality	
						Survivor	Non-survivor	Survivor	Non-survivor		%	Time of measurement
Han 2017	Korea	2011–2013	ADVIA 2120	286	Acute kidney injury with sepsis	59.6 ± 14.9	61.7 ± 14.6	65.6	62.0	Unclear	67.1	28 days
Kim 2017	Korea	2010–2011	ADVIA 2120	461	Acute cholangitis with septic shock	66.1 ± 13.1	70.7 ± 11.2	55.0	52.9	Immediately at ED admission	3.7	28 days
Kim 2014	Korea	2012–2011	ADVIA 2120	172	Gram negative bacteraemia	67.0 (15.0)	67.0 (16.0)	42.6	64.7	24 h from the onset of bacteraemia	9.9	10 days
Lim 2014	Korea	2010–2012	ADVIA 2120	75	Spontaneous bacterial peritonitis with sepsis	59.0 (38.0–82.0)^b,c^		87.7^c^		Prior to the administration of antibiotics	25.3	30 days
Hwang 2015	Korea	2012	ADVIA 2120	120	Sepsis	66.0 ± 14.1	68.2 ± 11.7	44.7	52.9	Immediately at ED admission	14.2	28 days
Kim 2012	Korea	2009–2010	ADVIA 120	102	Bacteraemia	64.0 ± 13.0	68.0 ± 18.0	55.1	50.0	72 h from the onset of bacteraemia	23.5	28 days
Seok 2012	Korea	2010	ADVIA 2120	129	Sepsis, severe sepsis^d^	64.0 (60.0–69.0)^c^		51.3^c^		Within 48 h of the onset of SIRS symptoms	24.8	28 days
Zanaty 2012	Egypt	N/R^d^	ADVIA 2120	53	Sepsis	58.6 ± 14.5	64.4 ± 12.4	69.4	76.5	Within the first 6 h of ICU admission	32.1	In hospital
Kim 2011^e^	Korea	2007–2010	ADVIA 120	116	Sepsis	69.3 ± 12.0^c^		N/R		N/R	15.5	N/R
Shin 2011^e^	Korea	2009–2010	N/R	71	Sepsis	63.0 ± 15.7^c^		63.6^c^		Unclear	78.9	N/R
Nahm 2008	Korea	N/R	ADVIA 120	237	Sepsis	55.4 ± 22.6	59.5 ± 22.9	N/R		N/R	35.9	28 days

^a^Age was presented as median (interquartile range) or mean ± standard deviation.

^b^Value of median (range).

^c^Value of total population.

^d^Seok *et al*. determined that Severe sepsis showed signs of organ dysfunction, hypoperfusion, metabolic acidosis, neurologic disorders, and septic shock.

^e^Abstract-only publication.

*Abbreviations*: DNI, delta neutrophil index; N/R, not reported; ED, emergency department; SIRS, systemic inflammatory response syndrome; ICU, intensive care unit.

### Quality of the included studies

In five of the nine studies (among which methodological quality were conducted), the quality criteria were fulfilled and they were deemed to be of high quality; the other four did not meet at least one criterion. Three studies were considered of low-quality. Details of our assessment of the quality are presented as Supplementary Figs S1 and S2. Additionally, all four studies which were included to measure the predictive accuracy of DNI for mortality were considered to be of high-quality (Supplementary Figs S3 and S4).

### Main analysis

**Figure 2:**
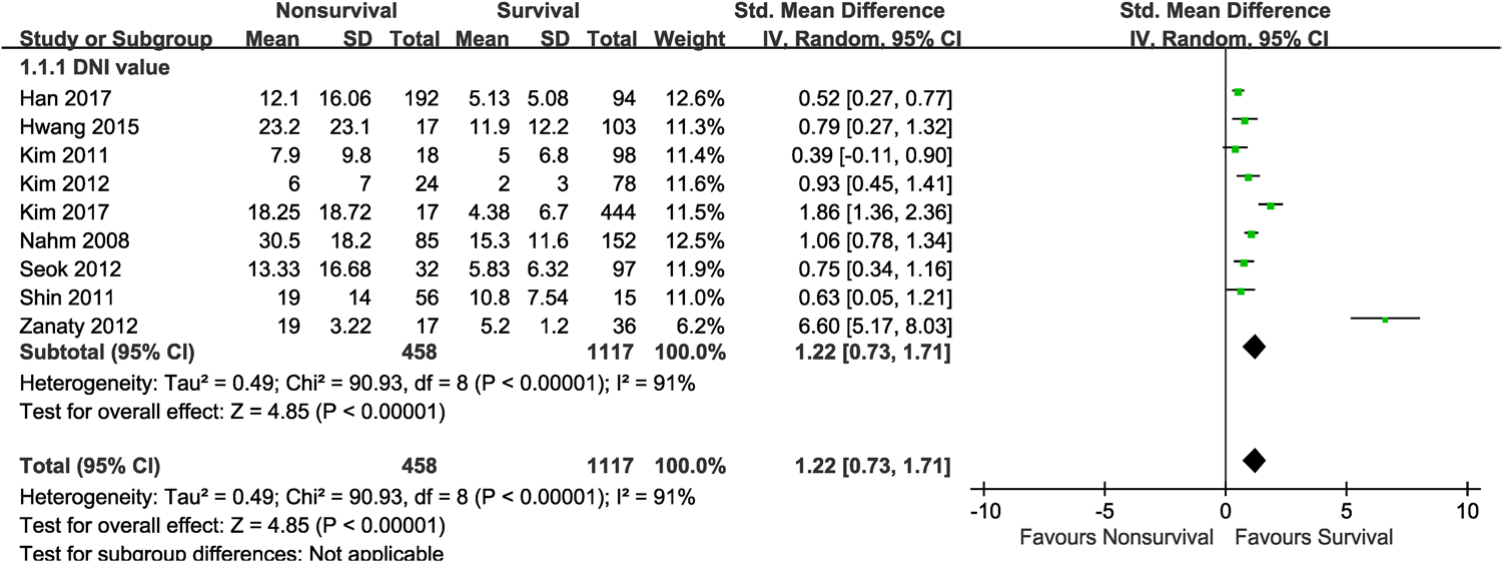
Meta-analysis for relevant studies. Mean delta neutrophil index value between non-survivors and survivors.

Eleven relevant studies including 1,822 patients were analysed. All of these studies reported differences in DNI values between survivors and non-survivors. In our meta-analysis, the DNI level was found to be significantly higher in non-survivors than in survivors, demonstrating a positive association with an overall SMD [(mean level in the non-survivor group – mean level in the survivor group)/pooled SD] of 1.22 (95% CI 0.73–1.71; I^2^ = 91%; *p* < 0.00001, Fig. 2). After excluding the two abstract-only publications, the SMD of the remaining seven studies was 1.46 (95% CI 0.86–2.06; I^2^ = 93%; *p* < 0.00001).

### Subgroup analysis and sensitivity analysis

**Figure 3:**
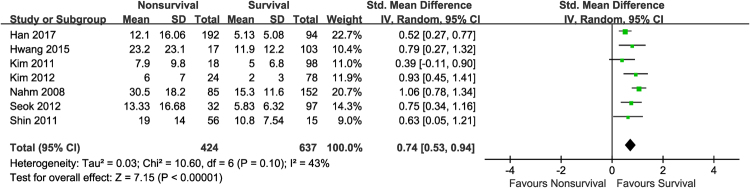
Sensitivity analysis; Forest plot of seven studies after removing the studies by Zanaty *et al*.^20^ and Kim *et al*.^23^.

We performed subgroup analyses according to the inclusion criteria, country of study, sample size, time of outcome assessment, and quality of included studies (Table 2). In the analysis for inclusion criteria, the SMD of the DNI was 1.28 (95% CI 0.73–16.08) and I^2^ was 92%. In the analysis for time of outcome assessment, the SMD was 1.61 (95% CI 0.83–2.39) and I^2^ was 80%. In the subgroup analyses, no item had low heterogeneity. A sensitivity analysis was performed by sequential removal of individual studies to minimize heterogeneity among the remaining studies. By removing the studies by Zanaty *et al*.^20^ and Kim *et al*.^23^, the heterogeneity was minimized to 43% (SMD 0.74, 95% CI 0.53–0.94; I^2^ = 43%, Fig. 3).

**Table 2:** Summary of standardized mean differences for mortality among subgroups.

Characteristic	Mortality			
	*N*	SMD (95% CI)	P value for heterogeneity	I^2^, %
All studies	9	1.22 (0.73, 1.71)	<0.00001	91
Inclusion criteria				
Bacteraemia	1	0.93 (0.45, 1.41)	—	—
Sepsis or septic shock	8	1.28 (0.73, 1.84)	<0.00001	92
Study location				
In Korea	8	0.86 (0.57, 1.15)	<0.00001	75
In Egypt	1	6.60 (5.17, 8.03)	—	—
Sample size				
≥100	7	0.89 (0.57, 1.21)	<0.0001	79
<100	2	3.58 (−2.28, 9.43)	<0.00001	98
Mortality assessment				
28-day	6	0.96 (0.62, 1.31)	0.0001	80
Other	3	2.40 (0.05, 4.75)	<0.00001	97
Study quality				
High	6	1.61 (0.83, 2.39)	<0.00001	94
Low	3	0.74 (0.30, 1.18)	0.05	66

*Abbreviations*: *N*, number; SMD, standardized mean difference; CI, confidence interval.

### Predictive accuracy of the DNI for mortality

**Figure 4:**
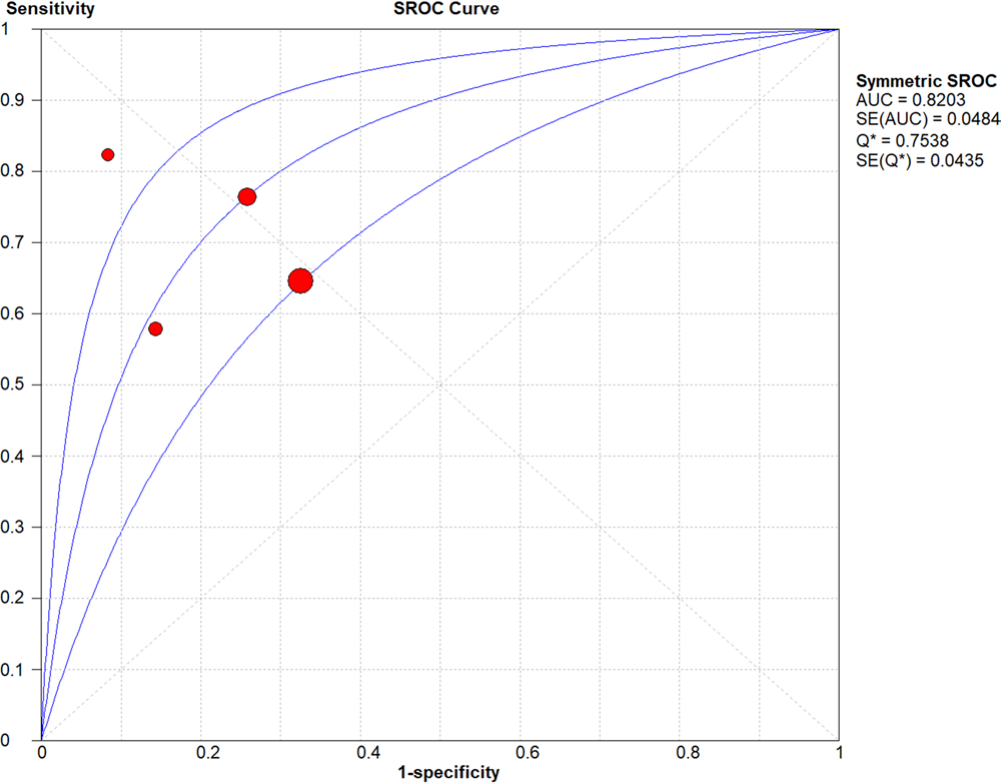
Summary receiver operating characteristic (SROC) curve of delta neutrophil index (DNI) for predicting mortality.

Meta-analysis was performed to compare the predictive accuracy of the DNI for mortality. The pooled area under the curve (AUC) based on thr summary receiver operating characteristic (SROC) curve was 0.82, which demonstrated a good grade of DNI in predicting mortality (Fig. 4).

Additionally, the pooled diagnostic odds ratio of DNI was 9.37 (95% CI 3.74–23.48) (Supplementary Table S2 and Fig. S5). Pooled sensitivity and specificity of DNI value were 0.70 (95% CI 0.60–0.80) and 0.72 (95% CI 0.68–0.75), respectively (Supplementary Table S2 and Fig. S6). Pooled positive and negative likelihood ratio were also 3.33 (95% CI 1.95–5.69) and 0.41 (95% CI 3.74–23.48), respectively (Supplementary Table S2). The best thresholds of DNI in predicting mortality were 1.3%, 5.2%, 5.7%, and 7.6% in each included study (Supplementary Table S2).

## Discussion

This systematic review and meta-analysis is the first to demonstrate that the DNI has prognostic value in adults with sepsis: High DNI values tended to be associated with mortality in septic patients. Since sepsis is a rapidly progressive and unpredictable disease regardless of the provision of appropriate treatment, the DNI could be a novel prognostic biomarker. Although the level of the DNI was significantly higher in non-survivors than in survivors, this meta-analysis showed high heterogeneity (SMD 1.22, 95% CI 0.73–1.71; I^2^ = 91%). To resolve this issue, subgroup analyses were performed for the clinical parameters thought to be inducing heterogeneity, such as the inclusion criteria used, country in which the studies were performed (Korea vs. Egypt), sample size (≥100 vs. <100), time of outcome assessment (28-day mortality vs. other), and quality of included studies (high vs. low). Despite these subgroup analyses, high levels of heterogeneity remained (Table 2). After performing an additional sensitivity analysis by removing studies by Zanaty *et al*.^20^ and Kim *et al*.^23^, heterogeneity was minimized (SMD 0.74, 95% CI 0.53–0.94; I^2^ = 43%, Fig. 3). The study by Zanaty *et al*.^20^ did not clearly explain the patient selection in the assessment of quality and had a relatively smaller sample size than the other included studies. In the study by Kim *et al*.^23^, the overall mortality in septic patients was exceptionally low at 3.7%; and much lower than that of the other included studies, whose mean estimate of overall mortality was 32.9%. We assumed that selection bias in the study by Zanaty *et al*.^20^ and the lower mortality rate in the study by Kim *et al*.^23^ contributed to the high heterogeneity in this meta-analysis.

Several confounding factors could have affected short-term mortality in this study. First, the use of appropriate antibiotics to treat the focus of infection in patients with sepsis influences mortality. Empiric broad-spectrum antibiotics should be administered to septic patients as soon as possible. In addition, the time to initiation of antibiotic administration is usually limited to 3–6 h^28–31^. After identification of a pathogen by blood culture, targeted antibiotic therapy should be initiated. However, in the included studies, data associated with antibiotic therapy were incomplete. Second, the severity of sepsis can affect mortality^32–34^. Previous studies have reported clinical severity scores, such as the acute physiology and chronic health evaluation (APACHE) and sequential organ failure assessment (SOFA) scores^35^. These scores can be used in subgroup analysis to resolve the high heterogeneity issue. However, information on severity scores could not be obtained from the included studies.

To diagnose sepsis early and predict mortality, procalcitonin is a promising biomarker that is widely used in adult patients. Although it is an excellent indicator of sepsis and has high sensitivity, there is controversy around its power to predict mortality^36,37^. Pettila *et al*. showed that procalcitonin values differ significantly between survivors and non-survivors^38^. In the present study, the DNI was not compared with the clinical prediction indices such as procalcitonin. Hence, the predictive power of DNI for mortality relative to these other indices remains unknown. Nevertheless, we found that the DNI has the potential to predict mortality in adults with sepsis. Thus, the DNI could be useful in rapidly identifying sepsis and determining whether early intervention to remove the septic focus should be performed. Further research to evaluate the use of the DNI in combination with other indices (such as procalcitonin) to increase predictive power in the overall assessment of sepsis, are warranted.

In a recent meta-analysis of the DNI by Park *et al*., the DNI was reported to have prognostic impact for mortality in septic patients^39^. The pooled sensitivity and specificity of the DNI for death were 0.70 (95% CI 0.56–0.81) and 0.78 (95% CI 0.73–0.83), respectively. The pooled AUC by SROC curve was also 0.84. However, in that meta-analysis, the same population with two different measures of mortality (10-day and 28-day mortality in the study by Kim, 2014) was included twice^39,40^. Additionally, inclusion of a paediatric study (Lee, 2013) contributed to heterogeneity in the population domain as all other included studies were performed with adult subjects^41^. Therefore, we thought that the prognostic value of the DNI in the meta-analysis by Park *et al*. was not appropriately evaluated. In our meta-analysis, we made an effort to perform a consistent analysis for a defined population (adult septic patients only) and a specific outcome (28-day mortality in the subgroup analysis). Our meta-analysis revealed the predictive accuracy of DNI for mortality in adult septic patients. The pooled specificity (0.72, 95% CI 0.68–0.75) and AUC (0.82) of the DNI for death were a little lower than that reported in the study by Park *et al*.^39^. We also found that the best threshold value of DNI in predicting mortality ranged from 1.3% to 7.6%.

The DNI is an unfamiliar blood marker; it evaluates infection by calculating the proportion of immature granulocytes. Although the leucocyte count is commonly and widely used to evaluate inflammation in infectious diseases, it can be affected by inflammation in non-infectious disorders such as rheumatoid arthritis, lupus, and malignancy^42–44^. In evaluating septic conditions, the DNI is therefore more valuable than the leucocyte count as it reflects the circulating immature granulocyte count irrespective of the leucocyte count^45^. Additionally, several studies have found that the DNI has the advantages of accuracy and rapidity in evaluating infection^22,23,39^. Hence, although the DNI is not widely used, these characteristics and advantages motivated us to evaluate further its value as a clinical prognostic biomarker.

There were several limitations in this study. First, wide representation was not secured because most included studies were geographically confined to South Korea. The findings of this study might have been different had patients from other countries with different health care systems or ethnicities been included. Additionally, all studies were single-centre investigations, reducing the generalisability of this study’s findings, which may therefore not be applied to most patients with sepsis. Additional studies with wider representation are required to yield more robust conclusions. Second, the pooled outcomes of this study were limited to short-term mortality. None of the included studies presented long-term (6-month or 1-year) mortality rates. Therefore, further studies are required to evaluate the effect of the DNI as a prognostic factor for long-term mortality in adults with sepsis. In conclusion, our systematic review and meta-analysis found that high DNI values tend to be associated with mortality in septic patients.

## Methods

### Search strategy and data sources

Using the Cochrane review methods^46^, we performed an extensive database search for studies evaluating the prognostic significance of the delta neutrophil index (DNI) in adult patients with sepsis. The literature search was performed by two experienced reviewers (Ahn C and Kim W) on November 21, 2017. We searched MEDLINE, EMBASE, and the Cochrane Library without language restrictions. Additionally, we checked the references of eligible studies to find related studies. Search keywords were selected following a discussion among all authors; the words decided on were: delta neutrophil, sepsis, systemic inflammatory response syndrome (see Supplementary Table S3).

### Study selection

All identified studies were inputted into Endnote 7.5 reference management software (Thomson Reuters, New York, NY, USA). Two reviewers (Ahn C and Kim W) independently selected all studies on the basis of predefined selection criteria. The title, abstract, and type of each identified article were checked in the screening stage. Duplicate articles were excluded after comparing the title, authors, and journal and year of publication of all identified studies. We obtained and assessed the full text of all potentially relevant studies in Portable Document Format electronic file format. Ultimately, included studies had the following features: (1) they involved adult patients with sepsis, (2) they included a measurement of the DNI; and (3) survival outcomes (28-day or in-hospital mortality) were assessed.

### Data extraction

Three reviewers (Ahn C, Kim W, and Lim TH) independently extracted the characteristics and outcomes of patients in the included studies. Discrepancies between reviewers were discussed and resolved by consensus. The following variables were extracted: the first author’s name, year of publication, country in which the study was conducted, inclusion period, equipment used for DNI measurement, study population, inclusion criteria, mortality, and mean (±SD) DNI level. If the latter was not available, estimated mean (±SD) levels were calculated from median values with interquartile ranges using the method of Wan *et al*.^47^. If any of these variables were not described in the studies, we sent relevant questions to the corresponding authors via email.

### Assessment of methodological quality

The methodological quality of nine identified studies were independently assessed by Ahn C and Kim W with blinding to authorship and journal using the Quality in Prognosis Studies (QUIPS) tool, with values of 2, 1, and 0 considered to be low, unclear, and high risk, respectively^48^. Studies achieving more than nine points from the sum of each six-item score were considered to be of high quality. Any unresolved disagreements between reviewers were resolved by discussion or review by the third author. Publication bias was not assessable in these studies. As tests for funnel plot asymmetry are generally only performed when at least 10 studies are included in a meta-analysis, this was not done in the present study.

Additionally, the methodological quality of four identified studies which include a predictive accuracy for mortality were assessed using the Quality Assessment of Diagnostic Accuracy Studies 2 (QUADAS-2) tool^49^.

### Statistical analysis

In the main analysis, we investigated the association between the initial DNI level and mortality among patients with sepsis. The strength of association between DNI and death was measured using the mean with SD between survivors and non-survivors, using a random effects model. DNI levels across comparison groups were extracted as mean differences with 95% CIs. To estimate heterogeneity, we estimated the proportion of between-study inconsistency due to the true differences between studies (rather than differences due to random error or chance) using the I^2^ statistic, with values of 25%, 50%, and 75% considered to be low, moderate, and high, respectively^50^. We conducted planned subgroup analyses based on inclusion criteria (sepsis/septic shock or bacteraemia); country (Korea or other); sample size (≥100 or <100 subjects); the time window of mortality assessment (within 28 days or other); the time window of DNI level measurement (within 24 h or other); and methodological quality of the study (high or low). SROC curve was used to predict mortality in adult septic patients, which also represented the calculated value of Q* index and AUC. The value of AUC was assessed using the following four AUC categories: more than 0.97 (excellent), from 0.93 to 0.96 (very good), from 0.75 to 0.92 (good), and less than 0.75 (reasonable but obviously deficient in prognostic accuracy)^51^. We used Review Manager version 5.3 (Cochrane Collaboration, Oxford, UK) to perform the statistical analysis, and a *P*-value < 0.05 was considered statistically significant.

### Data availability

The datasets generated and analysed during the current study are available from the corresponding author on reasonable request.

## Electronic supplementary material

Supplementary figures and tables

## References

[1] Dellinger RP (2013). Surviving Sepsis Campaign: international guidelines for management of severe sepsis and septic shock, 2012. Intensive Care Med..

[2] Garnacho-Montero J (2003). Impact of adequate empirical antibiotic therapy on the outcome of patients admitted to the intensive care unit with sepsis. Crit. Care Med..

[3] Singer M (2016). The third international consensus definitions for sepsis and septic shock (sepsis-3). JAMA..

[4] Liu D, Su L, Han G, Yan P, Xie L (2015). Prognostic value of procalcitonin in adult patients with sepsis: a systematic review and meta-analysis. PLoS One.

[5] Wacker C, Prkno A, Brunkhorst FM, Schlattmann P (2013). Procalcitonin as a diagnostic marker for sepsis: a systematic review and meta-analysis. Lancet Infect. Dis..

[6] Kim H, Kim Y, Lee HK, Kim KH, Yeo CD (2014). Comparison of the delta neutrophil index with procalcitonin and C-reactive protein in sepsis. Clin. Lab..

[7] Behnes M (2014). Diagnostic and prognostic utility of soluble CD 14 subtype (presepsin) for severe sepsis and septic shock during the first week of intensive care treatment. Crit. Care.

[8] Castelli GP (2004). Procalcitonin and C-reactive protein during systemic inflammatory response syndrome, sepsis and organ dysfunction. Crit. Care.

[9] Akpinar S, Rollas K, Alagöz A, Seğmen F, Sipit T (2014). Performance evaluation of MR-proadrenomedullin and other scoring systems in severe sepsis with pneumonia. J. Thorac. Dis..

[10] Vassiliou AG (2014). Elevated biomarkers of endothelial dysfunction/activation at ICU admission are associated with sepsis development. Cytokine.

[11] Cornbleet PJ (2002). Clinical utility of the band count. Clin. Lab. Med..

[12] Seebach JD, Morant R, Rüegg R, Seifert B, Fehr J (1997). The diagnostic value of the neutrophil left shift in predicting inflammatory and infectious disease. Am. J. Clin. Pathol..

[13] Cavallazzi R, Bennin C-L, Hirani A, Gilbert C, Marik PE (2010). Review of A Large Clinical Series: Is the Band Count Useful in the Diagnosis of Infection? An Accuracy Study in Critically Ill Patients. J. Intensive Care Med..

[14] Ha SO (2015). Fraction of immature granulocytes reflects severity but not mortality in sepsis. Scand. J. Clin. Lab. Invest..

[15] Mare TA (2015). The diagnostic and prognostic significance of monitoring blood levels of immature neutrophils in patients with systemic inflammation. Crit. Care.

[16] Ansari-Lari MA, Kickler TS, Borowitz MJ (2003). Immature granulocyte measurement using the Sysmex XE-2100: relationship to infection and sepsis. Am. J. Clin. Pathol..

[17] Seok Y (2012). Delta neutrophil index: a promising diagnostic and prognostic marker for sepsis. Shock.

[18] Nahm CH, Choi JW, Lee J (2008). Delta neutrophil index in automated immature granulocyte counts for assessing disease severity of patients with sepsis. Ann. Clin. Lab. Sci..

[19] Kim HW (2012). Delta neutrophil index: could it predict mortality in patients with bacteraemia?. Scand. J. Infect. Dis..

[20] Zanaty OM, Megahed M, Demerdash H, Swelem R (2012). Delta neutrophil index versus lactate clearance: Early markers for outcome prediction in septic shock patients. Alex. J. Med..

[21] Hwang YJ (2015). Newly designed delta neutrophil index–to–serum albumin ratio prognosis of early mortality in severe sepsis. Am. J. Emerg. Med..

[22] Han IM (2017). Delta neutrophil index is an independent predictor of mortality in septic acute kidney injury patients treated with continuous renal replacement therapy. BMC Nephrol..

[23] Kim H (2017). Usefulness of the delta neutrophil index as a promising prognostic marker of acute cholangitis in emergency departments. Shock.

[24] Kim HW (2014). Delta neutrophil index as a prognostic marker of early mortality in gram negative bacteremia. Infect. Chemother..

[25] Lim TS (2014). Use of the delta neutrophil index as a prognostic factor of mortality in patients with spontaneous bacterial peritonitis: Implications of a simple and useful marker. PLoS One.

[26] Kim HY (2011). Clinical significance of delta neutrophil index in patients with sepsis. Clin. Microbiol. Infect..

[27] Shin HJ (2011). Usefulness of delta neutrophil index (DNI) for SIRS patient in emergency department. Crit. Care Med..

[28] Ferrer R (2014). Empiric antibiotic treatment reduces mortality in severe sepsis and septic shock from the first hour: results from a guideline-based performance improvement program. Crit. Care Med..

[29] Ferrer R (2009). Effectiveness of treatments for severe sepsis: a prospective, multicenter, observational study. Am. J. Respir. Crit. Care Med..

[30] Rivers E (2012). Early interventions in severe sepsis and septic shock: a review of the evidence one decade later. Minerva Anestesiol..

[31] Gaieski DF (2010). Impact of time to antibiotics on survival in patients with severe sepsis or septic shock in whom early goal-directed therapy was initiated in the emergency department. Crit. Care Med..

[32] Mikkelsen ME (2009). Serum lactate is associated with mortality in severe sepsis independent of organ failure and shock. Crit. Care Med..

[33] Angus DC (2001). Epidemiology of severe sepsis in the United States: analysis of incidence, outcome, and associated costs of care. Crit. Care Med..

[34] Wichmann M, Inthorn D, Andress H-J, Schildberg F (2000). Incidence and mortality of severe sepsis in surgical intensive care patients: the influence of patient gender on disease process and outcome. Intensive Care Med..

[35] Rhodes A (2015). The surviving sepsis campaign bundles and outcome: results from the international multicentre prevalence study on sepsis (the IMPreSS study). Intensive Care Med..

[36] Kibe S, Adams K, Barlow G (2011). Diagnostic and prognostic biomarkers of sepsis in critical care. J. Antimicrob. Chemother..

[37] Ruiz-Alvarez MJ (2009). Diagnostic efficacy and prognostic value of serum procalcitonin concentration in patients with suspected sepsis. J. Intensive Care Med..

[38] Pettilä V, Hynninen M, Takkunen O, Kuusela P, Valtonen M (2002). Predictive value of procalcitonin and interleukin 6 in critically ill patients with suspected sepsis. Intensive Care Med..

[39] Park JH (2017). Delta neutrophil index (DNI) as a novel diagnostic and prognostic marker of infection: a systematic review and meta-analysis. Inflamm. Res..

[40] Kim HW (2014). Delta neutrophil index as a prognostic marker of early mortality in gram negative bacteremia. Infect. Chemother..

[41] Lee SM (2013). Usefulness of the delta neutrophil index for assessing neonatal sepsis. Acta Paediatr..

[42] Abramson N, Melton B (2000). Leukocytosis: basics of clinical assessment. Am. Fam. Physician.

[43] Pyo JY (2013). Delta neutrophil index as a marker for differential diagnosis between flare and infection in febrile systemic lupus erythematosus patients. Lupus.

[44] Pyo JY (2017). Delta neutrophil index contributes to the differential diagnosis between acute gout attack and cellulitis within 24 hours after hospitalization. Rheumatology (Oxford).

[45] Shin DH, Kim EJ, Kim SJ, Park J-Y, Oh J (2015). Delta neutrophil index as a marker for differential diagnosis between acute graft pyelonephritis and acute graft rejection. PLoS One.

[46] Higgins, J. P. & Green, S. *Cochrane handbook for systematic reviews of interventions. Version 5.1.0*. (The Nordic Cochrane Centre, Copenhagen, 2011).

[47] Wan X, Wang W, Liu J, Tong T (2014). Estimating the sample mean and standard deviation from the sample size, median, range and/or interquartile range. BMC Med. Res. Methodol..

[48] Hayden JA, van der Windt DA, Cartwright JL, Côté P, Bombardier C (2013). Assessing bias in studies of prognostic factors. Ann. Intern. Med..

[49] Whiting PF (2011). Quadas-2: A revised tool for the quality assessment of diagnostic accuracy studies. Ann. Intern. Med..

[50] Higgins J, Thompson SG (2002). Quantifying heterogeneity in a meta‐analysis. Stat. Med..

[51] Jones CM, Athanasiou T (2005). Summary Receiver Operating Characteristic Curve Analysis Techniques in the Evaluation of Diagnostic Tests. Ann. Thorac. Surg..

