# Supplementary material for: The delta neutrophil index (DNI) as a prognostic marker for mortality in adults with sepsis: a systematic review and meta-analysis
Source: Sci Rep. 2018 Apr 26;8:6621. doi: 10.1038/s41598-018-24211-7 (PMC5919925; doi:10.1038/s41598-018-24211-7)
Supplement: Supplementary file 1 — Supplementary figures and tables [file 41598_2018_24211_MOESM1_ESM.docx]

**The delta neutrophil index (DNI) as a prognostic marker for mortality in adults with sepsis: a systematic review and meta-analysis**

Chiwon Ahn^1,2,†^, Wonhee Kim^2,3,†^, Tae Ho Lim^4,*^, Youngsuk Cho^2,3^, Kyu-Sun Choi^5^, Bohyoung Jang^6^

^1^Department of Emergency Medicine, Armed Forces Yangju Hospital, Yangju, Korea
^2^Department of Biomedical Engineering, Graduate School of Medicine, Hanyang University, Seoul, Korea
^3^Department of Emergency Medicine, College of Medicine, Hallym University, Chuncheon, Korea
^4^Department of Emergency Medicine, College of Medicine, Hanyang University, Seoul, Korea
^5^Department of Neurosurgery, College of Medicine, Hanyang University, Seoul, Korea
^6^Department of Preventive Medicine, College of Korean Medicine, Kyung Hee University, Seoul, Korea

^†^Ahn and Kim contributed equally to this work.

**^*^Corresponding author**: Tae Ho Lim, M.D., Ph.D.
Department of Emergency Medicine, College of Medicine, Hanyang University, Seoul, Korea
222, Wangsimni-ro, Seongdong-gu, Seoul, 04763, Korea
Tel: +82-2-2290-8999
Fax: +82-2-2290-9280
E-mail: erthim@gmail.com

**Supplementary Figure S1**. Graph of risk of bias for this meta-analysis (QUIPS tool)


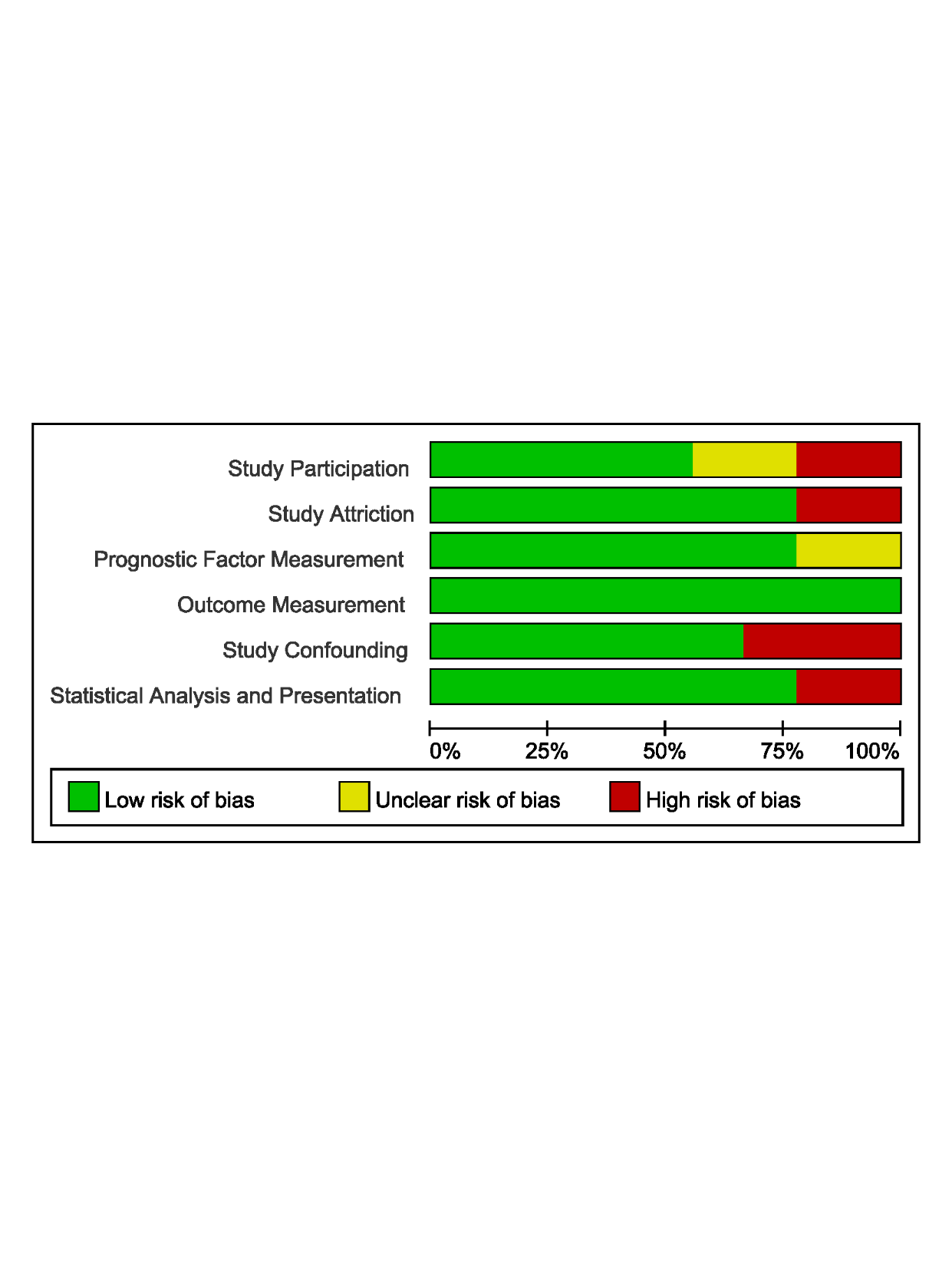


**Supplementary Figure S2.** Summary of the risk of bias of each study included in this meta-analysis (QUIPS tool)


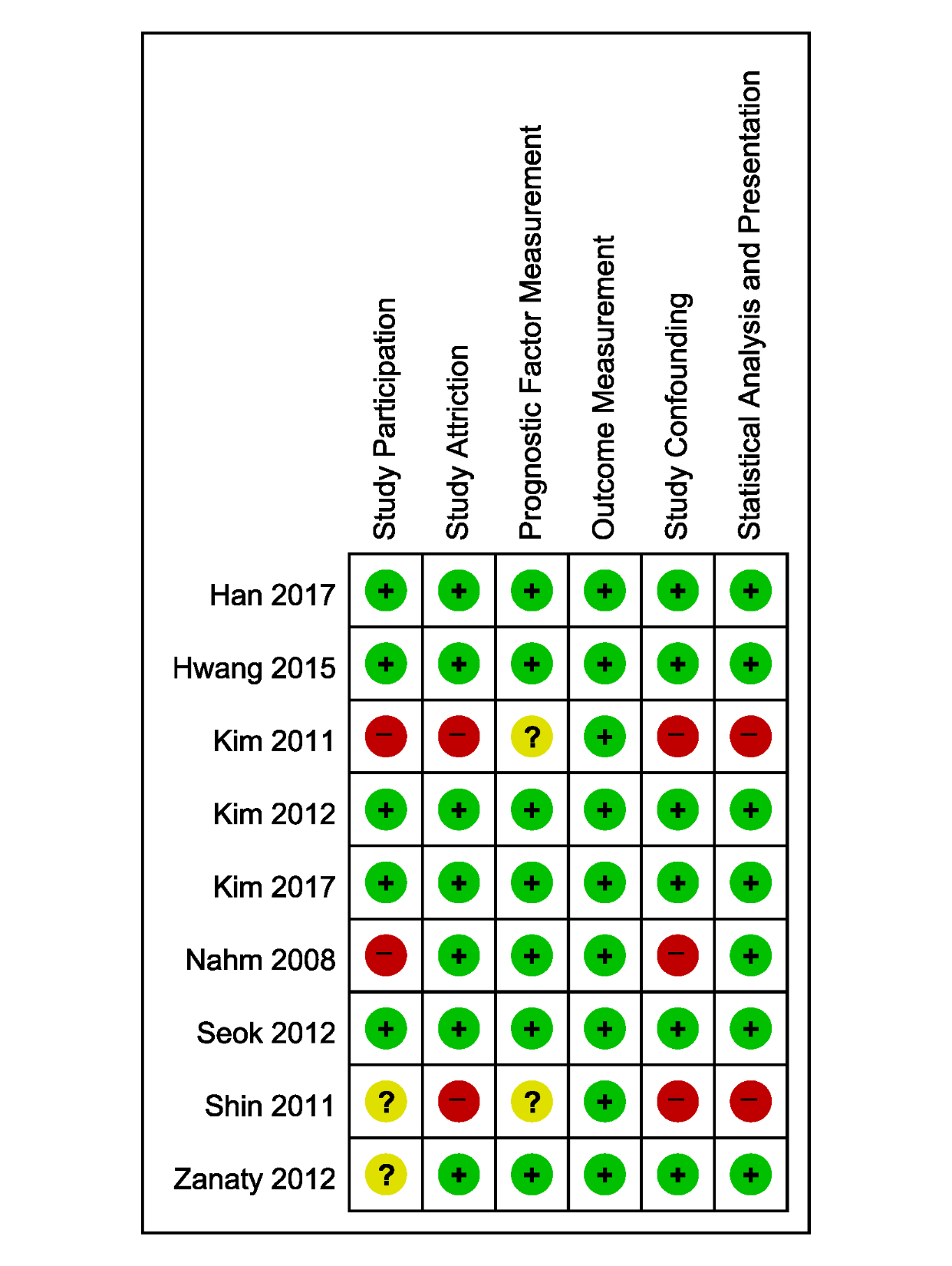


**Supplementary Figure S3**. Graph of risk of bias for this meta-analysis (QUADAS2 tool)


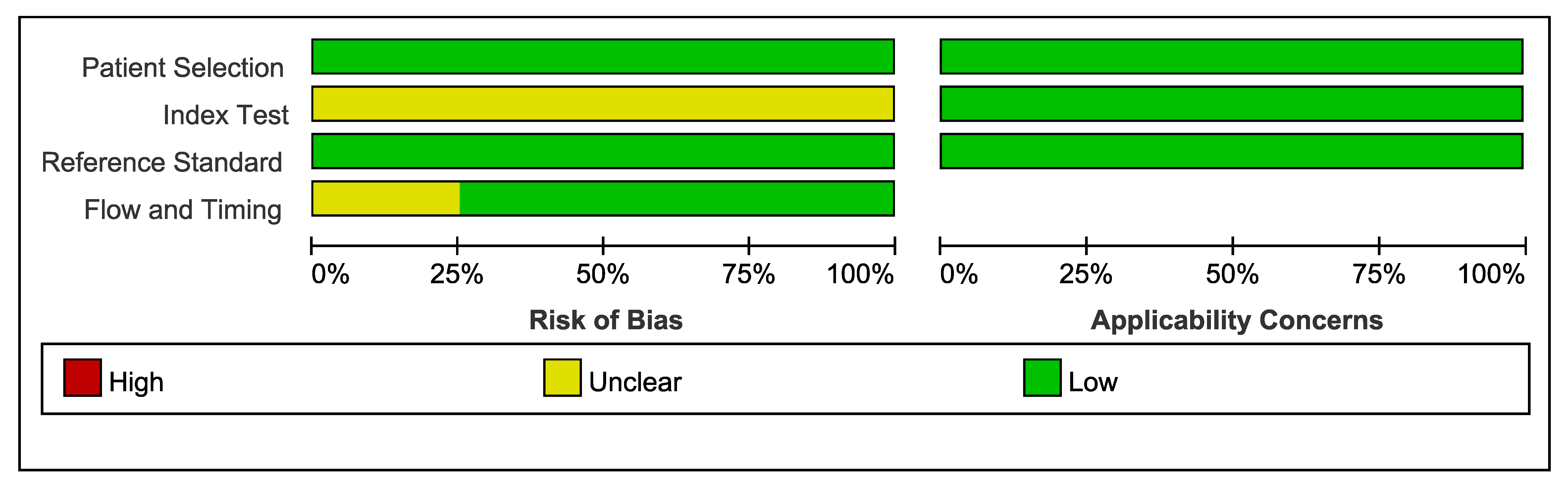


**Supplementary Figure S4.** Summary of the risk of bias of each study included in this meta-analysis (QUADAS2 tool)


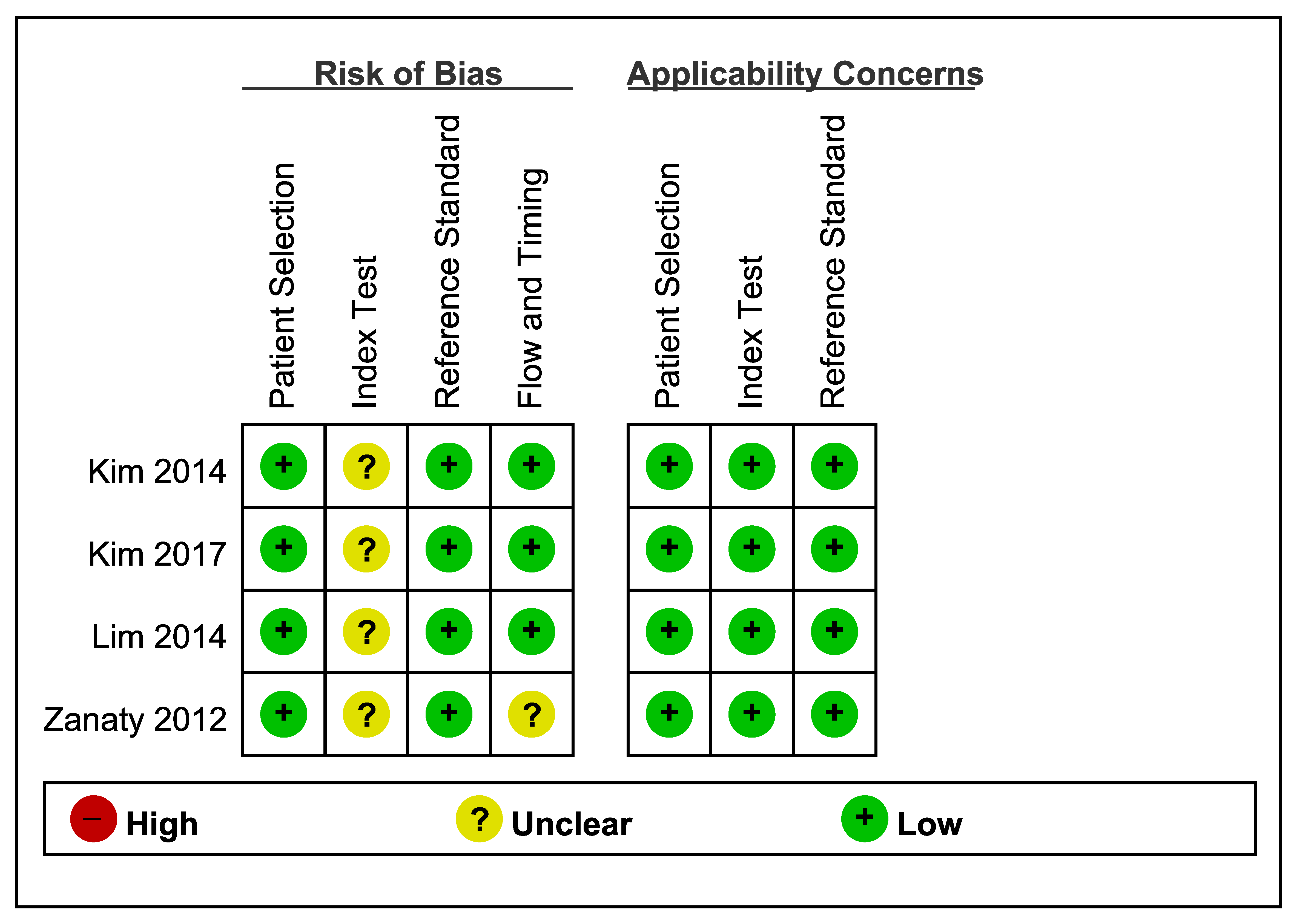


**Supplementary Figure S5.** Diagnostic odds ratio of delta neutrophil index for mortality


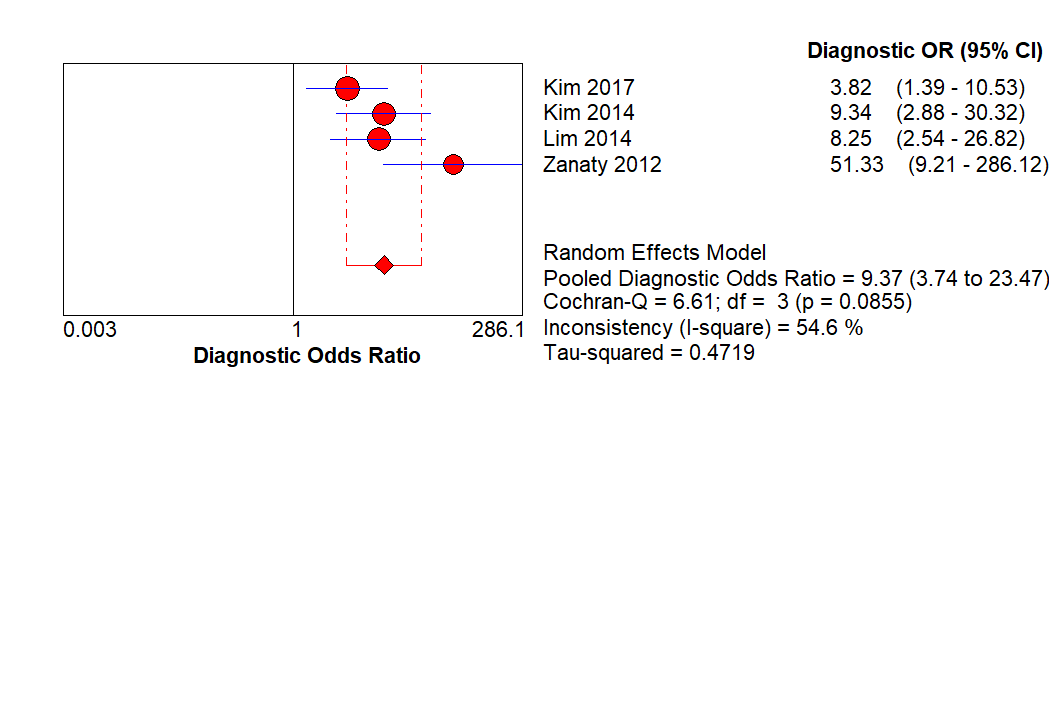


**Supplementary Figure S6.** Sensitivity and specificity of delta neutrophil index for mortality


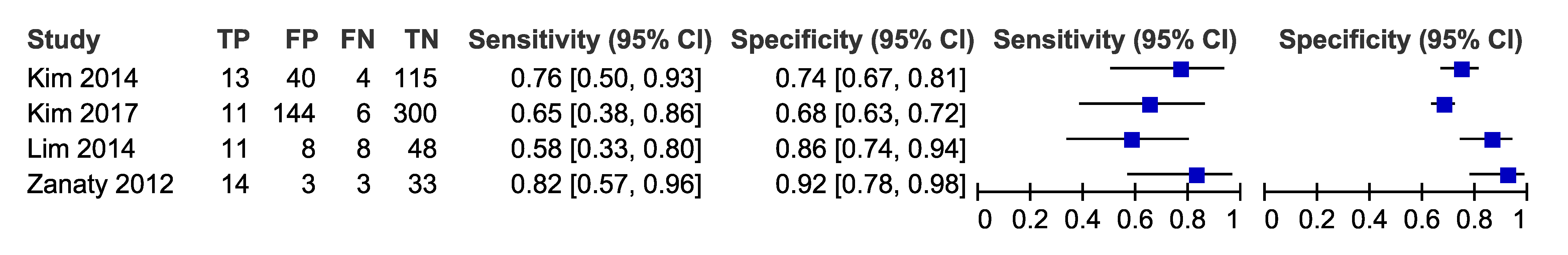


**Supplementary Table S1.** Detailed information of population

| Study | Site of infection site (%) | | | Blood culture (%) | Underlying disease (%) | | | | | | | Severity Score |
| --- | --- | --- | --- | --- | --- | --- | --- | --- | --- | --- | --- | --- |
|  | Lung | Abdomen | Genito-urinary tract |  | HTN | DM | Lung disease | Liver disease | CVD | Malignancy | Renal disease |  |
| Han 2017 | - | - | - | - | 48.9 / 33.9 | 42.6 / 30.2 | - | - | - | - | - | APACHE II  (24.0±7.5 / 28.1±7.3) SOFA  (10.8±3.7 / 13.1±2.0) |
| Kim 2017 | - | - | - | 41.89 / 82.35 | - | - | - | 6.76 / 17.65 | 0.68 / 0 | 27.48 / 70.59 | 0.45 / 0 | - |
| Hwang 2015 | - | - | - | - | 51.46 / 76.47 | 37.86 / 41.18 | 6.8 / 5.88 | 4.85 / 11.17 | 28.16 / 29.41 | 27.18 / 41.18 |  | - |
| Kim 2012 | 12.8 / 12.5 | 29.5 / 29.2 | 19.2 / 12.5 | 38.5 / 29.2 | 42.3 / 45.8 | 34.6 / 25 | -  - | 5.1 / 4.2 | -  - | 42.3 / 33.3 | 14.1 / 12.5 | - |
| Seok 2012^a^ | 14.1 | 16.7 | 23.1 | - | - | - | - | 0 | 5.1 | 52.6 | 6.4 | - |
| Zanaty 2012 | 52.8 / 64.7 | 22.2 / 23.5 | 19.4 / 11.8 | 47.2 / 70.6 | - | - | - | - | - | - | - | APACHE  (19.9±6.8 / 21.6±6.8) SOFA  (7.9±4.2 / 11.7±4.8) |
| Kim 2011 | - | - | - | 38.8 | - | - | - | - | - | - | - | APACHE  (total 17.8±6.9) |
| Shin 2011 | - | - | - | - | - | - | - | - | - | - | - | - |
| Nahm 2008 | - | - | - | - | - | - | - | - | - | - | - | - |

Each domain is described as a value corresponding to survivors / non-survivors.

^a^Results of the total population of survivors and non-survivors.

*Abbreviations*: HTN, hypertension; DM, diabetes mellitus; CVD, cardiovascular disease; APACHE, Acute Physiology and Chronic Health Evaluation; SOFA, Sequential Organ Failure Assessment.

**Supplementary Table S2.** Summary estimates of delta neutrophil index predicting mortality

| Study | Best threshold, % | Sensitivity (95% CI) | Specificity (95% CI) | Positive likelihood ratio | | Negative likelihood ratio | | DOR (95%CI) | |
| --- | --- | --- | --- | --- | --- | --- | --- | --- | --- |
|  |  |  |  | Ratio (95%, CI) | Weight, % | Ratio (95%, CI) | Weight, % |  | Weight, % |
| Kim 2017  Kim 2014  Lim 2014  Zanaty 2012 | 1.3  7.6  5.7  5.2 | 0.65 (0.38-0.86)  0.77 (0.50-0.93)  0.58 (0.34-0.80)  0.82 (0.57-0.96) | 0.68 (0.63-0.72)  0.74 (0.67-0.81)  0.86 (0.74-0.94)  0.92 (0.78-0.98) | 2.00 (1.37-2.91)  2.96 (2.04-4.31)  4.05 (1.92-8.56)  9.88 (3.27-29.85) | 31.85  31.87  21.78  14.49 | 0.52 (0.27-1.00)  0.32 (0.13-0.75)  0.49 (0.29-0.84)  0.19 (0.07-0.54) | 29.48  18.15  39.22  13.15 | 3.82 (1.39-10.53)  9.34 (2.88-30.32)  8.25 (2.54-26.82)  51.33 (9.21-286.10) | 29.65  26.35  26.31  17.69 |
| 4 studies | N/A | 0.70 (0.58-0.80) | 0.72 (0.68-0.75) | 3.33 (1.95-5.69) | N/A | 0.41 (0.28-0.60) | N/A | 9.37 (3.74-23.48) | N/A |
| Inconsistency (I-square,I^2^), % | N/A | 5.9 | 84.4 | 72.8 | | 16.0 | | 54.6 | N/A |

*Abbreviations*: CI, confidence interval; DOR, diagnostic odds ratio; N/A, not available

**Supplementary Table S3.** Comprehensive list presenting the search strategy

| Database | Search strategy |
| --- | --- |
| EMBASE | 1. ‘delta neutrophil’.mp. 75  2. exp ‘sepsis’/ 226,516  3. exp ‘bacterial infection’/ 838,000  4. ‘septic’.mp. 86,980  5. exp ‘systemic inflammatory response syndrome’/ 232,333  6. ‘SIRS’.mp. 8,093  7. or/2-6 1,021,433  8. 1 and 7 46 |
| MEDLINE | 1. ‘delta neutrophil’.mp. 32  2. exp ‘Sepsis’/ 117,844  3. exp ‘Bacterial Infection’/ 871,562  4. ‘septic’.mp. 57,366  5. exp ‘Systemic Inflammatory Response Syndrome’/ 121,730  6. ‘SIRS’.mp. 4,461  7. or/2-6 966,036  8. 1 and 7 20 |
| Cochrane | #1. delta neutrophil 37  #2. sepsis 8,634  #3. bacterial infection 11,603  #4. Septic 3,239  #5. systemic inflammatory response syndrome 1,530  #6. SIRS 419  #7. #2 or #3 or #4 or #5 or #6 21,495  #8. #1 and #7 13 |

The literature search was performed on November 21, 2017.
